# Impact of antagonistic muscle co-contraction on in vivo knee contact forces

**DOI:** 10.1186/s12984-018-0434-3

**Published:** 2018-11-08

**Authors:** Adam Trepczynski, Ines Kutzner, Verena Schwachmeyer, Markus O. Heller, Tilman Pfitzner, Georg N. Duda

**Affiliations:** 1 Julius Wolff Institute, Charité – Universitätsmedizin Berlin, corporate member of Freie Universität Berlin, Humboldt-Universität zu Berlin, and Berlin Institute of Health, Augustenburger Platz 1, 13353 Berlin, Germany; 2 Bioengineering Sciences Research Group, Faculty of Engineering and Physical Sciences, University of Southampton, Southampton, UK; 3 Center for Musculoskeletal Surgery, Charité – Universitätsmedizin Berlin, corporate member of Freie Universität Berlin, Humboldt-Universität zu Berlin, and Berlin Institute of Health, Berlin, Germany; 4 Clinic for Adult Hip and Knee Reconstruction, Vivantes Spandau Hospital, Berlin, Germany

**Keywords:** Muscle co-contraction, Musculoskeletal loading conditions; in vivo joint forces, Knee osteoarthritis

## Abstract

**Background:**

The onset and progression of osteoarthritis, but also the wear and loosening of the components of an artificial joint, are commonly associated with mechanical overloading of the structures. Knowledge of the mechanical forces acting at the joints, together with an understanding of the key factors that can alter them, are critical to develop effective treatments for restoring joint function. While static anatomy is usually the clinical focus, less is known about the impact of dynamic factors, such as individual muscle recruitment, on joint contact forces.

**Methods:**

In this study, instrumented knee implants provided accurate in vivo tibio-femoral contact forces in a unique cohort of 9 patients, which were used as input for subject specific musculoskeletal models, to quantify the individual muscle forces during walking and stair negotiation.

**Results:**

Even between patients with a very similar self-selected gait speed, the total tibio-femoral peak forces varied 1.7-fold, but had only weak correlation with static alignment (varus/valgus). In some patients, muscle co-contraction of quadriceps and gastrocnemii during walking added up to 1 bodyweight (~ 50%) to the peak tibio-femoral contact force during late stance. The greatest impact of co-contraction was observed in the late stance phase of stair ascent, with an increase of the peak tibio-femoral contact force by up to 1.7 bodyweight (66%).

**Conclusions:**

Treatment of diseased and failed joints should therefore not only be restricted to anatomical reconstruction of static limb axes alignment. The dynamic activation of muscles, as a key modifier of lower limb biomechanics, should also be taken into account and thus also represents a promising target for restoring function, patient mobility, and preventing future joint failure.

**Trial registration:**

German Clinical Trials Register: ID: DRKS00000606, date: 05.11.2010.

**Electronic supplementary material:**

The online version of this article (10.1186/s12984-018-0434-3) contains supplementary material, which is available to authorized users.

## Background

Joint-related disability represents a substantial global health burden and is a frequent consequence of osteoarthritis (OA), in which bone shape is altered and cartilage is lost, causing pain and preventing smooth motion of the joints. OA and failure of total joint replacement (TJR), later in life, are often thought to be caused by excessive mechanical loading [1]. Key determinants of joint loading in clinical assessment are measures of the skeletal anatomy, typically obtained from static radiographs, such as the angle between the long axes of the tibia and femur. Here, the varus/valgus angle quantifies whether a patient has bow-legged (varus) or knock-legged (valgus) knees, and the extent of varus/valgus mal-alignment [2]. It has been suggested that deviations from ideal knee alignment result in local mechanical overload and eventual joint failure, which explains the historical focus on restoring physiologically normal alignment [3]. However, to understand the mechanical failure of joints, it is essential to unravel the interplay of bones and other passive structures with the active muscles, which together create the musculoskeletal (MS) loading conditions that eventually lead to extreme joint contact forces (JCFs) [4, 5].

The most direct knowledge regarding JCFs is provided by in vivo measurements with instrumented joint implants [6–9], which show substantial intra-individual differences in the peak joint loads [7, 9]. Muscle forces dominate JCFs [10], and the variability of JCF magnitudes during the same task may thus result from variations in the structure of the muscle-tendon units, but may also arise from variations in muscle strength, muscle recruitment patterns, or the presence and extent of co-contraction of antagonist muscle groups. OA patients show increased levels of co-contraction compared to healthy controls [11–13], which may result from the disease, but could also be its primary cause, or at least a factor driving its progression [14]. Even after OA was treated with knee arthroplasty, increased levels of co-contraction have been reported [15], and related to muscular insufficiencies [16]. Co-contraction can also occur during rehabilitation such as during functional electrical stimulation, and lead to underestimation of the forces produced by the stimulated muscles in analyses based on the total muscle torque [17].

**Fig. 1:**
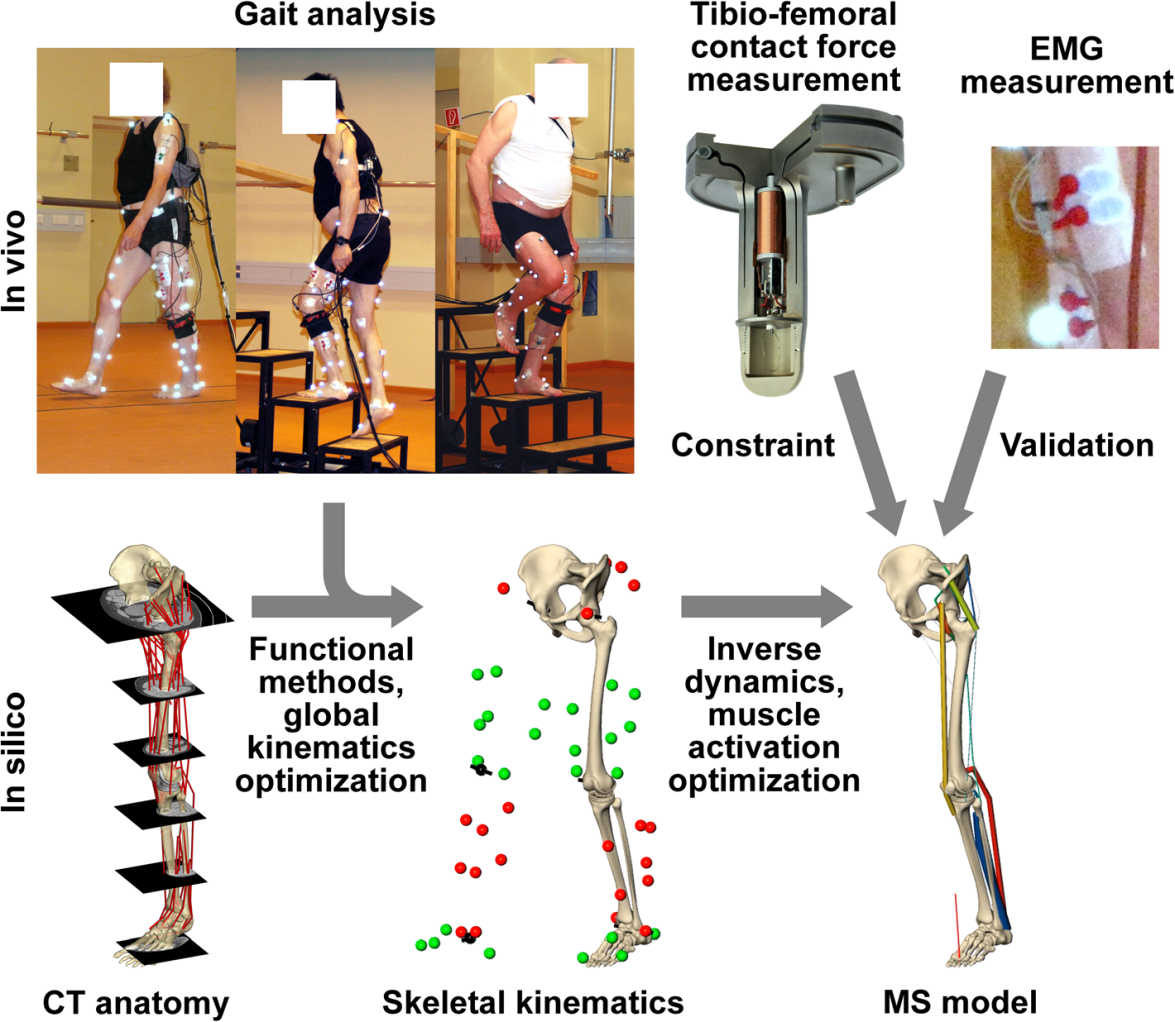
Overview of the methodology employed to assess internal loading, by combining measurements and musculoskeletal modelling. Gait analyses were performed during walking and stair negotiation, with simultaneous measurement of the in vivo tibio-femoral forces and surface EMG of the main muscles. Patient specific skeletal anatomy from CT was used to adapt reference muscles geometries, and then combined with functionally determined joint centres/axes to obtain the skeletal kinematics. The resulting musculoskeletal models were constrained to match the in vivo forces and verified using the EMG measurements

Musculoskeletal models can be used to predict all muscle forces and joint loading conditions, but require extensive validation against in vivo data [18]. While musculoskeletal models have been validated against in vivo measured data in a smaller cohort before [19–23], we are not aware of evidence suggesting that such models can consistently capture the variation in potentially sub-optimal muscle activation. Therefore, to obtain a reliable quantitative description of antagonist muscle co-contraction, we constrained personalized musculoskeletal models with synchronized tibio-femoral contact forces (TFCF), measured in vivo in a unique cohort of 9 patients with instrumented knee implants (Fig. 1) [24]. We then used the constrained musculoskeletal models to identify and quantify how muscle recruitment impacts joint contact forces, and thereby modulates them from minimizing the sum of squared muscle stresses – a criterion which best matched the in vivo forces among eight commonly utilized optimization criteria [20, 23, 25–29].

## Materials and methods

### Subjects and gait analysis

This study uses data from in vivo measurements of knee contact forces previously reported [10, 30]. Gait analyses were performed on 9 TKR patients (6 male, aged 70 ± 5 years, body mass 90.5 ± 12.6 kg, body height 1.72 ± 0.04 m), assessed 26 ± 13 months post-operatively (Fig. 1). Each subject obtained an instrumented knee implant based on a clinically proven design (INNEX, Zimmer GmbH, Winterthur, Switzerland) (Fig. 1) [24]. The instrumented implant allowed for in vivo measurement of the 3 force and 3 moment components of the contact load acting on the tibial tray (at approximately 100 Hz). The magnitude of the resultant JCF was calculated and is referred to as “in vivo TFCF” in this article. The ground reaction forces were captured by 2 force plates (AMTI, Watertown, USA) at 960 Hz, while 3D kinematics of the lower limbs were measured at 120 Hz using reflective markers (Vicon, Oxford, UK). Within a single measurement three activities were investigated: level walking, stair ascent (20 cm step height, 26 cm step run) and stair descent. Across all nine subjects, a total of 169 trials were captured. A standardized protocol with the same order of activities allowed for sufficient rest periods to prevent fatigue.

From post-operative CT data (with in plane resolution 0.4–0.5 mm and slice thickness 1–2 mm), three parameters characterizing the positioning of the tibial component were determined: the tilt of the tibial component in the coronal plane relative to the long axis of the tibia (range 3.4° valgus to 4.5° varus), the component’s posterior slope (3.1° to 11.4°), and its rotation (11.9° internal to 7.6° external). The mechanical axis angle (MAA), defined as the angle between the tibial and femoral mechanical axes, was derived from static coronal plane X-rays (4.5 valgus to 7.0° varus). In order to investigate the influence of alignment on the internal loads at the knee joint, the peak in vivo TFCF of each trial compared to the signed and absolute values of the aforementioned four static parameters using linear regression and one-way ANOVA (SPSS 22.0, IBM Corp., Armonk, NY). Here, the coefficient of determination (R^2^) was used as a measure to quantify the variability in the internal forces explained by parameters of static limb alignment. The ANOVA *p*-value was used to examine the statistical significance of potential correlations.

### Musculoskeletal modelling

A detailed description of the employed musculoskeletal modelling approach can be found in an earlier article [23], hence only a short description follows. Patient-specific bone anatomies were derived from post-operative CT scans, and combined with 51 reference muscle lines of action and wrapping points based on the Visible Human dataset (Fig. 1). The subsequent musculoskeletal analysis was performed with custom code written in C++, using optimization routines from the KNITRO (active set algorithm) and NAG (e04ucc) libraries. Functional methods were used to localize the dynamic joint centres and axes as previously reported [31–34]. The patient-specific skeletons were then aligned with the functional joint centres and axes using an inverse kinematics approach, to derive the skeletal kinematics and the muscle lines of action for the leg with the instrumented knee implant (Fig. 1). The hip and the ankle joint had 3 rotational degrees of freedom (DoF), while the tibio-femoral (TF) joint used the distal femur geometry to constrain the 6 DoF transformation between tibia and femur. Patello-femoral (PF) kinematics were determined based on a geometrical model driven by the TF kinematics under the assumption of constant patellar tendon length [23].

The joints and their local coordinate systems of the patient specific skeleton were defined as follows: hip centre – the centre of a sphere fitted to the femoral head geometry; hip proximal/distal (P/D) axis – perpendicular to the plane formed by the anterior superior iliac spines and the midpoint between the posterior superior iliac spines; hip anterior/posterior (A/P) axis – perpendicular to P/D axis and the line though both hip centres, hip medio/lateral (M/L) axis – perpendicular to P/D and A/P hip axes; knee centre – midpoint between the femoral epicondyles; knee P/D axis – line through knee and hip centres; knee A/P axis – perpendicular to knee P/D axis and the line though both femoral epicondyles; knee M/L axis – perpendicular to P/D and A/P knee axes; ankle centre – centre of circle though three points placed along the trochlea tali; ankle P/D axis – line through ankle centre and intercondylar eminence; ankle A/P axis – perpendicular to ankle P/D axis and the line though both malleoli; ankle M/L axis – perpendicular to P/D and A/P ankle axes; leg axis - the line connecting the of hip and ankle centres.

Previous studies have shown that muscle moments balance only ~ 1/3 of the external knee adduction moment (EAM) during walking and stair climbing, while the remaining ~ 2/3 are balanced passively by the contact moment resulting from asymmetrical distribution of the axial load among the condyles [10]. To determine the required muscles moment the in the frontal plane, the joint reaction force from inverse dynamics was distributed among the condyles to balance as much of the EAM as possible. During the subsequent muscle optimization, the muscles moments were only required to account for the portion of the EAM, which could not be balanced by the medio-lateral distribution of the joint reaction force from inverse dynamics. To verify this approach, the total axial muscle force from optimization was split evenly among both condyles, and added to the joint reaction forces from inverse dynamics. When compared to in vivo measured the medio-lateral distribution out approach predicted the medial ratio (*F*_*med*_ / (*F*_*med*_ + *F*_*lat*_)) with an error of 0.08 ± 0.05 (mean ± SD) in the constrained model over all time points of stance phase.

In order to determine the optimization criterion that best reproduces peak in vivo TFCFs, the contact forces throughout the entire stance phase were computed using 8 commonly utilized optimization criteria [20, 23, 25–29]: sum of muscle forces, sum of muscle stresses (linear, squared, cubed for both), and sum of JCFs (linear and squared) at the hip, knee and ankle (Additional file 1: Figure S1). In order to make the model predictions consistent with the individual total muscle forces levels, the in vivo measured TFCF was introduced as boundary condition into the optimization by combining the minimisation of the **S**um of **M**uscle **S**tresses **S**quared (*SMSS*) with the minimization of the **E**rror in the **TF** contact force prediction (*ETF*):

$$ ETF=\left|{F}_{model}-{F}_{\mathrm{in}\ \mathrm{vivo}}\right| $$

yielding the new constrained optimization criterion (*COC*) as the weighted sum of the two components:

$$ COC= nSMSS+w\times {nETF}^2 $$

where *nSMSS* and *nETF* are normalized values of *SMSS* and *ETF*, while *w* is a weighting factor. The normalized values *nSMSS* and *nETF* were computed by dividing *SMSS* and *ETF* by their respective values that would result from a maximal contraction of all muscles. Note that for the ETF term the normalization factor is determined individually for each given time point. The choice of the value of *w* was informed by the maximal *ETF* within each trial, expressed as function of *w*: *ETF*_*max*_(*w*). For a value of *w* = 10 the mean slope of *ETF*_*max*_(*w*) for each activity and patient dropped to 5% or less of the initial slope at *w* = 0, meaning that a further increase of *w* would not reduce *ETF*_*max*_ in a relevant manner (Additional file 1: Figure S2). The difference between COC and SMSS in terms of TFCFs and individual muscle forces was computed for all three activities, at the two peaks of the in vivo TFCF.

Based on the muscle forces from the model, the amount of co-contraction was quantified based on the work of Rudolph et al. [35] by the co-contraction index (CCI) defined as:

$$ \mathrm{CCI}=\left({\mathrm{F}}_1+{\mathrm{F}}_2\right)\times \operatorname{MIN}\left({\mathrm{F}}_1,{\mathrm{F}}_2\right)/\operatorname{MAX}\left({\mathrm{F}}_1,{\mathrm{F}}_2\right) $$

where F_1_ and F_2_ are the total forces of two antagonistic muscle groups.

### EMG processing and validation of muscle activities

To validate the predicted muscle activation patterns against the muscle activation of the major knee muscles, EMG signals were recorded for six of the nine patients at 9600 Hz using a measurement system by Biovision (Wertheim, Germany) and processed using MATLAB (version 7.7.0, MathWorks, Inc., MA). The frequencies relevant to muscle activation (10-500 Hz) were extracted using a zero-phase Butterworth filter, after which the signals were rectified and smoothed using a two-pass sliding average with a 0.1 s window. The processed EMG signals were aggregated into 3 groups (vasti, gastrocnemii and hamstrings) by adding the medial and lateral signals within each group. The forces predicted by the *COC*-model were added similarly: vasti = vastus med. + vastus lat.; gastrocnemii = gastrocnemius med. + gastrocnemius lat.; hamstrings = semitendinosus + semimembranosus + biceps femoris. For a comparison between the resulting EMG envelopes and the muscle forces predicted by *COC*-model, the signal of each muscle group in both data sets was normalized to its maximum during all stance phases of the same patient and activity [36]. The activation state of the muscle groups was identified from the normalized data using a 50% threshold of the maximal value [37]. The temporal agreement was quantified as the fraction of the time during which the on/off state was consistent between EMG and the model.

## Results

### Weak correlations between static anatomy and in vivo peak tibio-femoral contact forces

Peak TFCF magnitudes varied substantially across the nine subjects. The ranges for the mean (±SD) peak TFCFs were 2.05 ± 0.10–3.48 ± 0.11 BW (bodyweight) for walking, 2.30 ± 0.06–4.39 ± 0.26 BW for stair ascent, and 2.95 ± 0.06–4.37 ± 0.21 BW for stair descent. Linear regression did not imply strong relationships between the mean in vivo peak TFCFs and any of the implantation angles of the tibial component or the mechanical axis angle (MAA) of the leg. The coefficient of determination (R^2^) was never higher than 0.3 and the ANOVA *p*-value was never below 0.14.

### Constraining the model with in vivo data

**Fig. 2:**
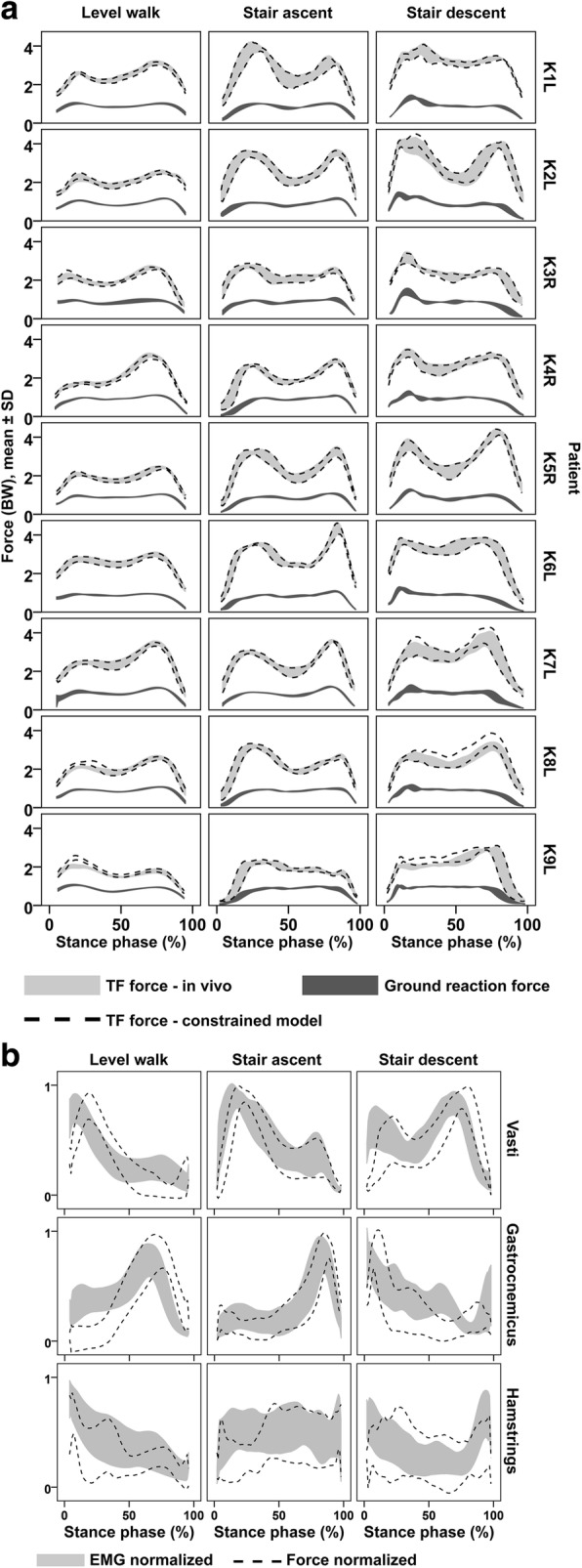
Comparison of constrained model results and measurements **a** Tibio-femoral forces measured in vivo (light grey), tibio-femoral forces from the constrained model (between dashed lines), and the measured ground reaction forces (dark grey).The indicated range covers the mean ± 1SD as determined from repeated trials. **b** Comparison of the EMG data (recorded in 6 of the 9 patients) to the computed muscle forces from the constrained model, normalized by the maximum of each patient and activity

Among the eight initially investigated optimization criteria, the best overall match of peak TFCF between model and measurement was achieved when the sum of squared muscle stresses was minimized (*SSMS*), with mean relative errors of the peak TFCF predictions (|*F*_*model*_ - *F*_in vivo_| / *F*_in vivo_) of 16 ± 14% for walking, 14 ± 11% for stair ascent and 13 ± 9% stair descent (Additional file 1: Figure S1). When minimization of squared muscle stresses was combined with matching the in vivo TFCF in the constrained optimization criterion (*COC*), these relative errors were reduced to 4 ± 6%, 1 ± 1% and 3 ± 4% of the in vivo TFCF respectively for walking, stair ascent and stair descent (Fig. 2a). The largest remaining errors occurred during walking for K9L (21 ± 5%) and stair descent for K8L (11 ± 3%). The predicted peak hip contact force changed from 3.63 ± 0.31BW, 3.46 ± 0.49BW and 3.46 ± 0.45BW for SSMS to 3.70 ± 0.28BW, 3.63 ± 0.54BW and 3.60 ± 0.49BW for *COC*, respectively for walking, stair ascent and stair descent.

For the six patients with available EMG data, the muscle activation profiles determined from *COC* were generally in good qualitative agreement with the EMG patterns (Fig. 2b). On average the activation states of the EMG and the model were in agreement 77% of the total stance phase. The weakest activation state agreement was observed for the hamstrings during stair ascent with 62 ± 8% (mean ± SD) of the time, ranging from 52 to 71% across patients. The best agreement was found for the gastrocnemii during stair ascent with 89 ± 6% of the time, ranging from 78 to 95% across patients (Table 1).

**Table 1:** The fraction of time during which the predicted muscle activation state matches the EMG derived activation state

		Fraction of time with activation match	
Muscle group	Activity	mean ± SD	range across patients
Vasti	Level walk	83 ± 06%	76–91%
	Stair ascent	82 ± 03%	79–86%
	Stair descent	66 ± 09%	57–83%
Gastrocnemii	Level walk	69 ± 08%	60–79%
	Stair ascent	89 ± 06%	78–95%
	Stair descent	80 ± 06%	72–90%
Hamstrings	Level walk	79 ± 09%	67–89%
	Stair ascent	62 ± 08%	52–71%
	Stair descent	69 ± 17%	54–92%

### Constrained muscle forces at the knee

**Fig. 3:**
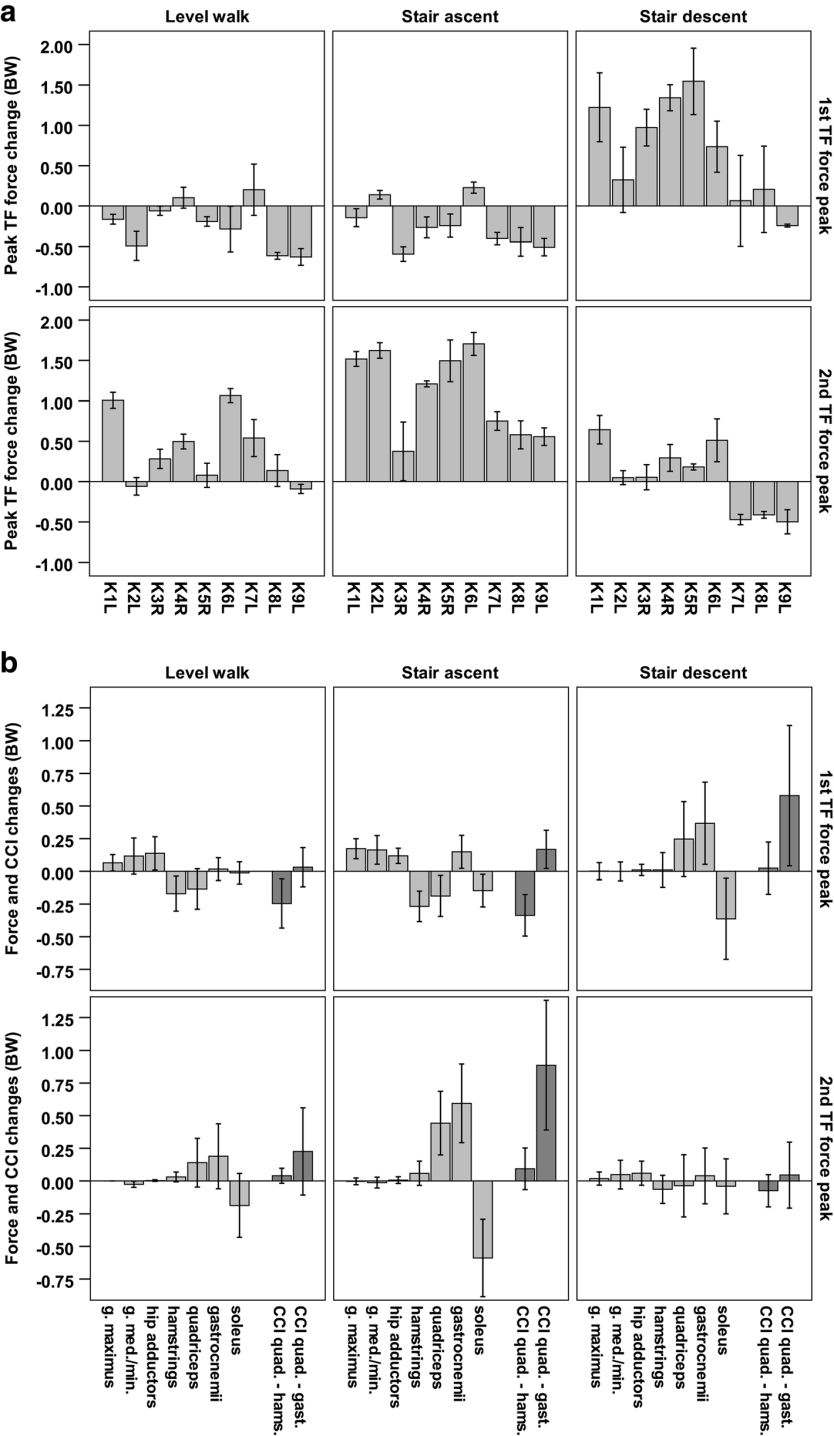
Changes in the internal forces introduced by constraining the model to match in vivo TF forces (COC), compared to minimizing the sum of muscle stresses squared (SMSS). **a** The changes in TFCF per patient. **b** The most relevant changes of forces by individual muscle groups (light grey), and the changes in co-contraction (CCI) of the knee flexors and extensors (dark grey), averaged for all patients (mean ± SD)

Compared to SMSS model the COC model increased TFCF by up to 1.70 ± 0.14BW (2nd peak of stair ascent, patient K6L), and reduced it by up to 0.63 ± 0.10BW (1st peak during walking, patient K9L). While TFCF decreased in most patients during the 1st peaks of walking and stair ascent, it usually increased at the other investigated four TF force peaks (Fig. 3a). The reductions of TFCF were mainly achieved by reducing the co-contraction of the quadriceps and the hamstrings by up to 0.59 ± 0.05BW (1st peak during stair ascent, patient K1L), which coincided with increased forces of the muscles crossing only the hip. The increases of TFCF were mainly due to an increase in co-contraction of the quadriceps and the gastrocnemii by up to 1.45 ± 0.31BW (2nd peak of stair ascent, patient K2L), which coincided with decreased soleus activity (Fig. 3b).

## Discussion

Degeneration of natural and failure of replaced joints is, among other factors, frequently associated with mechanical overloading [14, 38–41]. Frequently, the joint axis as seen in X-rays is assumed to be the key determinant of mechanical forces acting at the joint and surgical strategies are judged by the quality of a so called “mechanical” or “anatomical” axis alignment. However, the forces acting in vivo and their determinants are widely unknown. From instrumented implant measurements, the in vivo JCFs are known to be multiples of bodyweight, even during normal walking. Such high forces are the direct consequence of muscle activity contributing up to 3/4 of the total TFCF [10]. Substantial variation in the in vivo JCFs for a given physical task suggests a high variability, not only in muscle recruitment patterns but also in the total forces exerted by the muscles [7, 9]. However, previous attempts to understand the exact role of the muscles in mechanical overload have been challenged by the difficulty to quantify *in vivo* muscle forces. Whilst EMG measurements can provide information about the temporal activation patterns of a subset of superficial muscles, there is no trivial, direct link between muscle activation levels and muscle forces [42].

Musculoskeletal modelling can provide a prediction of joint and muscle forces, but such modelling approaches have not been shown to predict TF forces accurately across more than 2 subjects [23, 43]. The presented analyses confirm that conventional approaches to determine muscle forces, aimed at efficient muscle employment [26, 44], cannot predict the individual level of antagonist muscle co-contraction. In these conventional approaches, co-contraction is avoided as a suboptimal strategy, requiring alternative methods to predict muscle co-contraction [42, 45]. We therefore used the largest available group of 9 patients with instrumented knee implants [24] to constrain personalized musculoskeletal models, and to quantify the individual amount of muscle forces resulting from co-contraction during 3 activities of daily living. To our knowledge, this is the first time that *in vivo* contact forces and in silico modelling were matched for 9 patients and multiple activities with varying knee flexion ranges. The large range of in vivo measured TF contact forces [9] within our cohort, with 1.7-fold variation even at similar gait speeds, provides a significantly wider representation of inter-subject variability than in any previous analysis. Importantly, our analyses show that this TF force variability could not be explained with differences in MAA or tibial implantation alignment, parameters that have previously been a key focus of research and clinical management of knee OA [46, 47].

The comparison of the analyses constrained by the in vivo joint contact forces (COC) to analysis strategy using commonly employed optimality criteria to estimate JCFs (SMSS) revealed that additional muscle co-contraction can increase the peak of TFCF by up to 66% in late stance during stair ascent, and by up to 56% in late stance during walking (Fig. 3a). These increases of the TFCF were the result of up to ~1BW additional force produced by the co-contraction of the gastrocnemii and the quadriceps. The variations in joint contact forces of peak forces during walking provide an indication of how far off results computed under the assumption of “optimal” muscle activation can be from reality, and underlines the decisive role of co-contraction for JCFs. Moreover, the muscle activation patterns found when constraining the solution by the in vivo measured TFCF resulted in changes to the contact forces also across the hip joint. Failure to accurately capture the manner in which muscles are activated is therefore likely to results in errors in the internal musculoskeletal loading conditions throughout the entire lower limb. While the SMSS model prefers to activate the soleus during the late stance push off, the in vivo strategy in most subjects appears to be the synergetic use of the gastrocnemii and the quadriceps. Such a strategy could allow the quadriceps to indirectly contribute to the plantar flexion moment, at the cost of higher TFCF. On the other hand, the in vivo based model resulted in lower TFCFs during the early stance phase of walking and stair ascent, by employing less co-contraction of the hamstrings and the quadriceps than the SMSS model.

Current approaches to the treatment of joint injury and degeneration primarily focus on passive structures and their effect on load distribution and joint function [46, 47]. Our findings indicate that even an “optimally” reconstructed joint axis does not necessarily prevent mechanical overloading of the joint. To avoid such overloading, the muscle activity level and their control have to be taken into account. Antagonist muscle co-contraction can also reduce tensile stresses in bones under bending loads and thus reduce fracture risk [48]. Furthermore, while co-contraction does not contribute to balancing the external moment, it can substantially stiffen the joint and thus it more stable against unexpected changes in the external load [49, 50]. A need or desire for increased joint stability at the expense of movement efficiency may thus explain increased levels of co-contraction. How the individual muscle co-contraction can be assessed, and if an intra-operative ligament tensioning or balancing could help to adjust a patients’ individual soft tissue mechanics needs to be further researched. Also, it remains unclear why some subjects require higher levels of joint stabilization by muscle co-contraction, and how such individual systematic “overloading” of joints could be modulated or prevented.

The approach to quantify the muscle forces based on in vivo TFCFs as used in this study has certain limitations. Whilst the introduction of the in vivo TFCFs as boundary conditions to the optimization problem reduces the available solution space, and thereby eliminates a considerable proportion of solutions not consistent with the overall extent of muscle forces acting at the joint, there is no guarantee that the muscle activation pattern identified in this manner is indeed the very pattern utilized by a subject. However, we believe that using the optimization criterion with the least TFCF prediction error, and constraining it further to match the actual in vivo TFCF, provides the best currently possible estimation of muscle forces at the knee. Further studies which, additionally to the in vivo JCFs, also use comprehensive EMG measurements as boundary conditions to further constrain the solution space could provide even more realistic muscle force estimations [51]. While our small cohort of TKR patients cannot represent all habitual and cultural variations of human locomotion, it is still the largest group worldwide in which in vivo measurements of the TFCF are possible. Similarly to TKR patients, increased levels of co-contraction have also been previously reported in patients with advanced stages of OA [11–13, 15, 52, 53]. Despite its limited size, our cohort represents a wide range of TF loading levels and co-contraction, enabling an unprecedented quantitative analysis of the influence of muscle-co-contraction on internal loading conditions at the knee.

## Conclusions

In conclusion, we found that muscle forces dominate joint loading during dynamic activities, and that individual variations in antagonist muscle co-contraction can lead to substantially different JCFs. Treatment and rehabilitation of diseased and failed joints should thus not only consider the alignment of static limb axes, but also consider the dynamic activation of muscles as a promising new target for restoring function, patient mobility and preventing future joint failure. Computational models can be instrumental for realizing that vision, but need to consider the large variability in muscle activation strategies employed by TJR patients as demonstrated in this study.

## Additional file

Additional file 1: **Figure S1.** Tibio-femoral joint contact force (TFCF) prediction errors as function of the 9 optimization criteria used in the study. (Top) RMS errors (mean ± SD) of the prediction throughout the stance phase (top). (Bottom) Absolute difference of predicted and measured peak TFCFs. The constrained model (*COC*) uses the combination of squared muscle stresses and in vivo TFCFs, which was used to quantify the co-contraction. **Figure S2.** Maximal errors in TFCF prediction per trial (*ETF*_*max*,_ mean ± SD) as function of the weight w for the constraint enforcing the measured TFCF in the *COC* optimization criterion. The indicated *w* = 10 was the value at which the mean slope of *ETF*_*max*_(*w*) dropped to below 5% of its initial value at *w* = 0, and which was thus used in the subsequent analyses. At a value of w = 10 the mean ± SD of the maximum error of the TFCF was 0.16 ± 0.13BW for level walking, 0.10 ± 0.05BW for stair ascent and 0.29 ± 0.22BW for stair descent. (DOC 163 kb)

## Abbreviations

A/P: Anterior/posterior
BW: Bodyweight
COC: Constrained optimization criterion
DoF: Degree of freedom
EMG: Electromyography
F_med_ / F_med_: Medial/lateral axial tibio-femoral contact force
JCF: Joint contact forces
M/L: Medio/lateral
MAA: Mechanical axis angle
MS: Musculoskeletal
OA: Osteoarthritis
P/D: Proximal/distal
PF: Patello-femoral
SMSS: Sum of muscle stresses squared
TF: Tibio femoral
TFCF: Tibio-femoral contact forces
TJR: Total joint replacement

## References

[1] Andriacchi TP (2012). Osteoarthritis: Probing knee OA as a system responding to a stimulus. Nat Rev Rheumatol.

[2] Paley D. Principles of deformity correction: Springer Berlin Heidelberg; 2002.

[3] Kettelkamp DB, Chao EY (1972). A method for quantitative analysis of medial and lateral compression forces at the knee during standing. Clin Orthop Relat Res.

[4] Bennell KL, Wrigley TV, Hunt MA, Lim BW, Hinman RS (2013). Update on the role of muscle in the genesis and management of knee osteoarthritis. Rheum Dis Clin N Am.

[5] Wilson DR, McWalter EJ, Johnston JD (2008). The measurement of joint mechanics and their role in osteoarthritis genesis and progression. Rheum Dis Clin N Am.

[6] Bergmann G, Deuretzbacher G, Heller M, Graichen F, Rohlmann A, Strauss J (2001). Hip contact forces and gait patterns from routine activities. J Biomech.

[7] Damm P, Schwachmeyer V, Dymke J, Bender A, Bergmann G (2013). In vivo hip joint loads during three methods of walking with forearm crutches. Clin Biomech (Bristol, Avon)..

[8] D'Lima DD, Patil S, Steklov N, Slamin JE, Colwell CW (2006). Tibial forces measured in vivo after total knee arthroplasty. J Arthroplast.

[9] Kutzner I, Heinlein B, Graichen F, Bender A, Rohlmann A, Halder A (2010). Loading of the knee joint during activities of daily living measured in vivo in five subjects. J Biomech.

[10] Trepczynski A, Kutzner I, Bergmann G, Taylor WR, Heller MO (2014). Modulation of the relationship between external knee adduction moments and medial joint contact forces across subjects and activities. Arthritis Rheumatol.

[11] Hubley-Kozey CL, Hill NA, Rutherford DJ, Dunbar MJ, Stanish WD (2009). Co-activation differences in lower limb muscles between asymptomatic controls and those with varying degrees of knee osteoarthritis during walking (vol 24, pg 407, 2009). Clin Biomech.

[12] Heiden TL, Lloyd DG, Ackland TR (2009). Knee joint kinematics, kinetics and muscle co-contraction in knee osteoarthritis patient gait. Clin Biomech (Bristol, Avon)..

[13] Schmitt LC, Rudolph KS (2007). Influences on knee movement strategies during walking in persons with medial knee osteoarthritis. Arthritis Rheum.

[14] Felson DT (2013). Osteoarthritis as a disease of mechanics. Osteoarthr Cartil.

[15] Benedetti MG, Catani F, Bilotta TW, Marcacci M, Mariani E, Giannini S (2003). Muscle activation pattern and gait biomechanics after total knee replacement. Clin Biomech (Bristol, Avon).

[16] Yoshida Y, Mizner RL, Snyder-Mackler L (2013). Association between long-term quadriceps weakness and early walking muscle co-contraction after total knee arthroplasty. Knee.

[17] Levin O, Mizrahi J, Isakov E (2000). Transcutaneous FES of the paralyzed quadriceps: is knee torque affected by unintended activation of the hamstrings?. J Electromyogr Kinesiol.

[18] Hicks JL, Uchida TK, Seth A, Rajagopal A, Delp SL (2015). Is my model good enough? Best practices for verification and validation of musculoskeletal models and simulations of movement. J Biomech Eng.

[19] D'Lima DD, Fregly BJ, Patil S, Steklov N, Colwell CW (2012). Knee joint forces: prediction, measurement, and significance. P I Mech Eng H.

[20] Heller MO, Bergmann G, Deuretzbacher G, Dürselen L, Pohl M, Claes L (2001). Musculo-skeletal loading conditions at the hip during walking and stair climbing. J Biomech.

[21] Lin YC, Walter JP, Banks SA, Pandy MG, Fregly BJ (2010). Simultaneous prediction of muscle and contact forces in the knee during gait. J Biomech.

[22] Stansfield BW, Nicol AC, Paul JP, Kelly IG, Graichen F, Bergmann G (2003). Direct comparison of calculated hip joint contact forces with those measured using instrumented implants. An evaluation of a three-dimensional mathematical model of the lower limb. J Biomech.

[23] Trepczynski A, Kutzner I, Kornaropoulos E, Taylor WR, Duda GN, Bergmann G (2012). Patellofemoral joint contact forces during activities with high knee flexion. J Orthop Res.

[24] Heinlein B, Graichen F, Bender A, Rohlmann A, Bergmann G (2007). Design, calibration and pre-clinical testing of an instrumented tibial tray. J Biomech.

[25] Li G, Kaufman KR, Chao EY, Rubash HE (1999). Prediction of antagonistic muscle forces using inverse dynamic optimization during flexion/extension of the knee. J Biomech Eng.

[26] Crowninshield RD, Brand RA (1981). A physiologically based criterion of muscle force prediction in locomotion. J Biomech.

[27] Jackson M, Benkhemis I, Begon M, Sardain P, Vallee C, Lacouture P (2012). Identifying the criterion spontaneously minimized during the take-off phase of a sub-maximal long jump through optimal synthesis. Multibody Syst Dyn.

[28] Herzog W, Binding P (1993). Cocontraction of pairs of antagonistic muscles: analytical solution for planar static nonlinear optimization approaches. Math Biosci.

[29] Pedotti A, Krishnan VV, Stark L (1978). Optimization of muscle-force sequencing in human locomotion. Math Biosci.

[30] Kutzner I, Trepczynski A, Heller MO, Bergmann G (2013). Knee adduction moment and medial contact force--facts about their correlation during gait. PLoS One.

[31] Ehrig RM, Heller MO, Kratzenstein S, Duda GN, Trepczynski A, Taylor WR (2011). The SCoRE residual: a quality index to assess the accuracy of joint estimations. J Biomech.

[32] Ehrig RM, Taylor WR, Duda GN, Heller MO (2006). A survey of formal methods for determining the Centre of rotation of ball joints. J Biomech.

[33] Ehrig RM, Taylor WR, Duda GN, Heller MO (2007). A survey of formal methods for determining functional joint axes. J Biomech.

[34] Taylor WR, Kornaropoulos EI, Duda GN, Kratzenstein S, Ehrig RM, Arampatzis A (2010). Repeatability and reproducibility of OSSCA, a functional approach for assessing the kinematics of the lower limb. Gait Posture.

[35] Rudolph KS, Axe MJ, Snyder-Mackler L (2000). Dynamic stability after ACL injury: who can hop?. Knee Surg Sports Traumatol Arthrosc.

[36] Bolgla LA, Uhl TL (2007). Reliability of electromyographic normalization methods for evaluating the hip musculature. J Electromyogr Kinesiol.

[37] Howard RM, Conway R, Harrison AJ (2017). Muscle activation sequencing of leg muscles during linear glide shot putting. Sports Biomech.

[38] Bennell KL, Creaby MW, Wrigley TV, Bowles KA, Hinman RS, Cicuttini F (2010). Bone marrow lesions are related to dynamic knee loading in medial knee osteoarthritis. Ann Rheum Dis.

[39] Miyazaki T, Wada M, Kawahara H, Sato M, Baba H, Shimada S (2002). Dynamic load at baseline can predict radiographic disease progression in medial compartment knee osteoarthritis. Ann Rheum Dis.

[40] Neogi T, Nevitt M, Niu J, Sharma L, Roemer F, Guermazi A (2010). Subchondral bone attrition may be a reflection of compartment-specific mechanical load: the MOST study. Ann Rheum Dis.

[41] Wimmer MA, Andriacchi TP, Natarajan RN, Loos J, Karlhuber M, Petermann J (1998). A striated pattern of wear in ultrahigh-molecular-weight polyethylene components of miller-Galante total knee arthroplasty. J Arthroplast.

[42] Buchanan TS, Lloyd DG, Manal K, Besier TF (2004). Neuromusculoskeletal modeling: estimation of muscle forces and joint moments and movements from measurements of neural command. J Appl Biomech.

[43] Fregly BJ, Besier TF, Lloyd DG, Delp SL, Banks SA, Pandy MG (2012). Grand challenge competition to predict in vivo knee loads. J Orthop Res.

[44] Kaufman KR, An KW, Litchy WJ, Chao EY (1991). Physiological prediction of muscle forces--I. theoretical formulation. Neuroscience.

[45] Forster E, Simon U, Augat P, Claes L (2004). Extension of a state-of-the-art optimization criterion to predict co-contraction. J Biomech.

[46] Benjamin J (2006). Component alignment in total knee arthroplasty. Instr Course Lect.

[47] Sikorski JM (2008). Alignment in total knee replacement. J Bone Joint Surg British Vol.

[48] Mizrahi J, Verbitsky O, Isakov E (2000). Fatigue-related loading imbalance on the shank in running: a possible factor in stress fractures. Ann Biomed Eng.

[49] Hogan N (1984). Adaptive-control of mechanical impedance by Coactivation of antagonist muscles. Ieee T Automat Contr.

[50] Heitmann S, Ferns N, Breakspear M (2011). Muscle co-contraction modulates damping and joint stability in a three-link biomechanical limb. Front Neurorobot.

[51] Mizrahi J, Mizrahi J (2011). The role of electromyograms in resolving musculoskeletal interactions in able-bodied and disabled human individuals. Advances in applied electromyography: IntechOpen.

[52] Fallah-Yakhdani HR, Abbasi-Bafghi H, Meijer OG, Bruijn SM, van den Dikkenberg N, Benedetti MG (2012). Determinants of co-contraction during walking before and after arthroplasty for knee osteoarthritis. Clin Biomech (Bristol, Avon)..

[53] Zeni JA, Rudolph K, Higginson JS (2010). Alterations in quadriceps and hamstrings coordination in persons with medial compartment knee osteoarthritis. J Electromyogr Kinesiol.

