# Supplementary material for: Impact of antagonistic muscle co-contraction on in vivo knee contact forces
Source: J Neuroeng Rehabil. 2018 Nov 8;15:101. doi: 10.1186/s12984-018-0434-3 (PMC6225620; doi:10.1186/s12984-018-0434-3)
Supplement: Supplementary file 1 — Figure S1. Tibio-femoral joint contact force (TFCF) prediction errors as function of the 9 optimization criteria used in the study. (Top) RMS errors (mean ± SD) of the prediction throughout the stance phase (top). (Bottom) Absolute difference of predicted and measured peak TFCFs. The constrained model (COC) uses the combination of squared muscle stresses and in vivo TFCFs, which was used to quantify the co-contraction. Figure S2. Maximal errors in TFCF prediction per trial (ETFmax, mean ± SD) as function of the weight w for the constraint enforcing the measured TFCF in the COC optimization criterion. The indicated w = 10 was the value at which the mean slope of ETFmax(w) dropped to below 5% of its initial value at w = 0, and which was thus used in the subsequent analyses. At a value of w = 10 the mean ± SD of the maximum error of the TFCF was 0.16 ± 0.13BW for level walking, 0.10 ± 0.05BW for stair ascent and 0.29 ± 0.22BW for stair descent. (DOC 163 kb) [file 12984_2018_434_MOESM1_ESM.doc]

Additional file 1


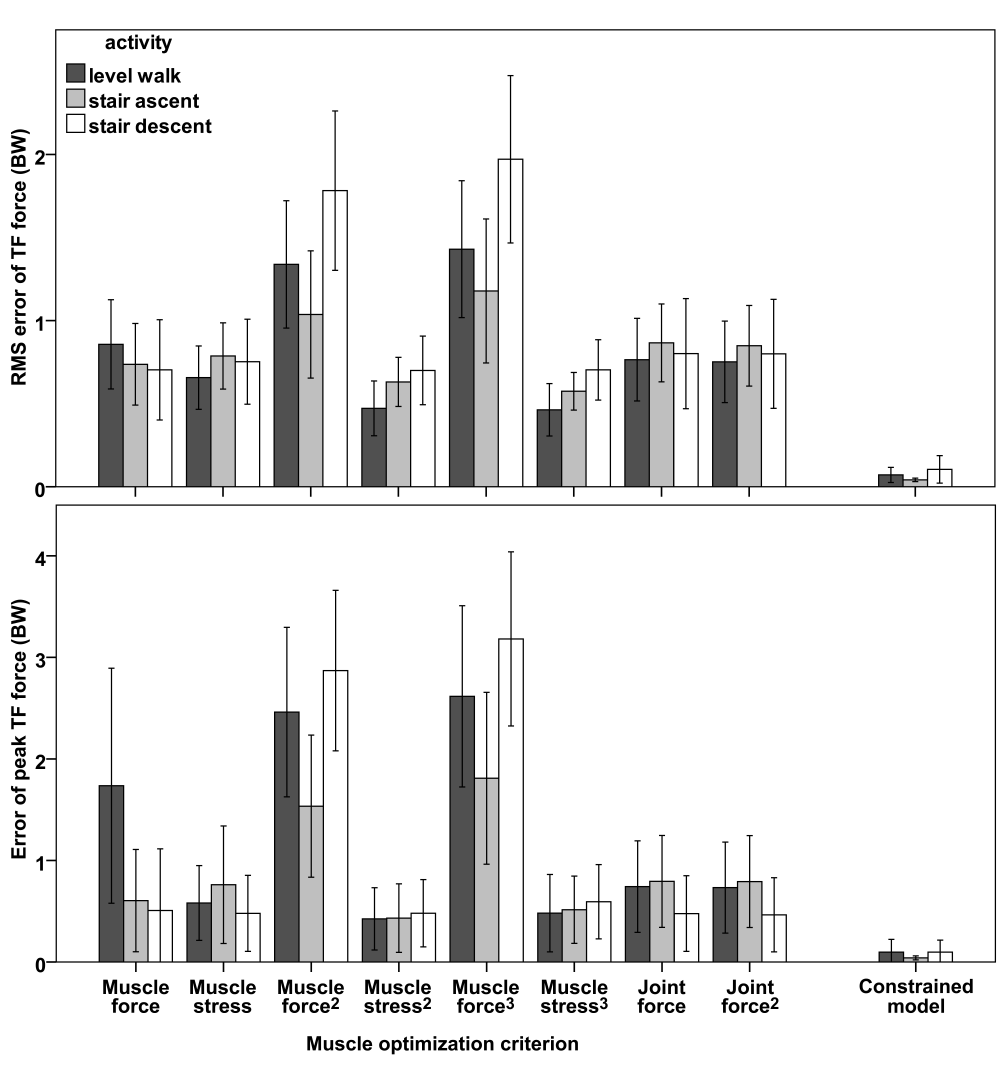


**Figure S1.** Tibio-femoral joint contact force (TFCF) prediction errors as function of the 9 optimization criteria used in the study. (Top) RMS errors (mean±SD) of the prediction throughout the stance phase (top). (Bottom) Absolute difference of predicted and measured peak TFCFs. The constrained model (*COC*) uses the combination of squared muscle stresses and *in vivo* TFCFs, which was used to quantify the co-contraction.


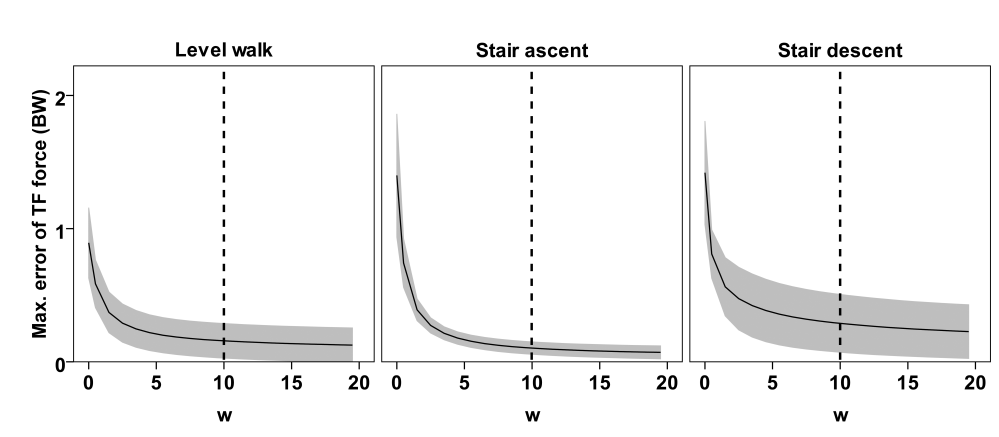


**Figure S2.** Maximal errors in TFCF prediction per trial (*ETF_max_*, mean±SD) as function of the weight w for the constraint enforcing the measured TFCF in the *COC* optimization criterion. The indicated *w*=10 was the value at which the mean slope of *ETF_max_*(*w*) dropped to below 5% of its initial value at *w*=0, and which was thus used in the subsequent analyses. At a value of w=10 the mean±SD of the maximum error of the TFCF was 0.16±0.13BW for level walking, 0.10±0.05BW for stair ascent and 0.29±0.22BW for stair descent.
